# Supplementary material for: Fast and accurate protein structure search with Foldseek
Source: Nat Biotechnol. 2023 May 8;42(2):243–6. doi: 10.1038/s41587-023-01773-0 (PMC10869269; doi:10.1038/s41587-023-01773-0)
Supplement: Supplementary file 1 — Supplementary Tables 1–4 and Supplementary Figs. 1–14. [file 41587_2023_1773_MOESM1_ESM.pdf]

nature biotechnology

Brief Communication

https://doi.org/10.1038/s41587-023-01773-0

Fast and accurate protein structure search with Foldseek

In the format provided by the authors and unedited

Fast and accurate protein structure search with Foldseek

Michel van Kempen[1,*], Stephanie S. Kim[2,*], Charlotte Tumescheit[2], Milot Mirdita[1], Jeongjae Lee[2], Cameron L.M. Gilchrist[2], Johannes Söding[1,3,†] and Martin Steinegger[2,4,5,†]

[1] Quantitative and Computational Biology, Max Planck Institute for Multidisciplinary Sciences, Göttingen, Germany. [2] School of Biological Sciences, Seoul National University, Seoul, South Korea. [4] Campus-Institute Data Science (CIDAS), Goldschmidtstrasse 1, 37077 Göttingen, Germany. [4] Artificial Intelligence Institute, Seoul National University, Seoul, South Korea [5] Institute of Molecular Biology and Genetics, Seoul National University, Seoul, 08826, South Korea[*] These authors contributed equally. [†] Authors to whom correspondence should be addressed.
Contact: soeding@mpinat.mpg.de, martin.steinegger@snu.ac.kr

Name	Mutual information [bit]
3Di	1.37
CLE	1.08
3D-BLAST	1.07

Supplementary Table 1: **Mutual information per aligned residue pair** To estimate the information density of each structural alphabet, we measured the average mutual information between two structurally aligned residues x and y. For this, we calculated the substitution frequencies for each alphabet as described in "3Di substitution score matrix". Then, the mutual information equals $\sum_{x,y=1}^{20} p(x,y)\, \log_2 \frac{p(x,y)}{p(x)\,p(y)}$. We note that the mutual information is an upper limit on the mutual information per residue in sequences, since consecutive residues depend on each other, lowering the information content of sequences and thereby lowering the mutual information between structurally aligned sequences. Since 3Di sequences have weaker dependencies between consecutive residues than the CLE and 3D-BLAST alphabets (not shown), the gain in mutual information of 3Di over the two other alphabets is even higher than shown here.

	A	C	D	E	F	G	H	I	K	L	M	N	P	Q	R	S	T	V	W	Y	X
A	6	-3	1	2	3	-2	-2	-7	-3	-3	-10	-5	-1	1	-4	-7	-5	-6	0	-2	0
C	-3	6	-2	-8	-5	-4	-4	-12	-13	1	-14	0	0	1	-1	0	-8	1	-7	-9	0
D	1	-2	4	-3	0	1	1	-3	-5	-4	-5	-2	1	-1	-1	-4	-2	-3	-2	-2	0
E	2	-8	-3	9	-2	-7	-4	-12	-10	-7	-17	-8	-6	-3	-8	-10	-10	-13	-6	-3	0
F	3	-5	0	-2	7	-3	-3	-5	1	-3	-9	-5	-2	2	-5	-8	-3	-7	4	-4	0
G	-2	-4	1	-7	-3	6	3	0	-7	-7	-1	-2	-2	-4	3	-3	4	-6	-4	-2	0
H	-2	-4	1	-4	-3	3	6	-4	-7	-6	-6	0	-1	-3	1	-3	-1	-5	-5	3	0
I	-7	-12	-3	-12	-5	0	-4	8	-5	-11	7	-7	-6	-6	-3	-9	6	-12	-5	-8	0
K	-3	-13	-5	-10	1	-7	-7	-5	9	-11	-8	-12	-6	-5	-9	-14	-5	-15	5	-8	0
L	-3	1	-4	-7	-3	-7	-6	-11	-11	6	-16	-3	-2	2	-4	-4	-9	0	-8	-9	0
M	-10	-14	-5	-17	-9	-1	-6	7	-8	-16	10	-9	-9	-10	-5	-10	3	-16	-6	-9	0
N	-5	0	-2	-8	-5	-2	0	-7	-12	-3	-9	7	0	-2	2	3	-4	0	-8	-5	0
P	-1	0	1	-6	-2	-2	-1	-6	-6	-2	-9	0	4	0	0	-2	-4	0	-4	-5	0
Q	1	1	-1	-3	2	-4	-3	-6	-5	2	-10	-2	0	5	-2	-4	-5	-1	-2	-5	0
R	-4	-1	-1	-8	-5	3	1	-3	-9	-4	-5	2	0	-2	6	2	0	-1	-6	-3	0
S	-7	0	-4	-10	-8	-3	-3	-9	-14	-4	-10	3	-2	-4	2	6	-6	0	-11	-9	0
T	-5	-8	-2	-10	-3	4	-1	6	-5	-9	3	-4	-4	-5	0	-6	8	-9	-5	-5	0
V	-6	1	-3	-13	-7	-6	-5	-12	-15	0	-16	0	0	-1	-1	0	-9	3	-10	-11	0
W	0	-7	-2	-6	4	-4	-5	-5	5	-8	-6	-8	-4	-2	-6	-11	-5	-10	8	-6	0
Y	-2	-9	-2	-3	-4	-2	3	-8	-8	-9	-9	-5	-5	-5	-3	-9	-5	-11	-6	9	0
X	0	0	0	0	0	0	0	0	0	0	0	0	0	0	0	0	0	0	0	0	0

Supplementary Table 2: **Substitution scores for 3Di states** The scores are log-odd scores in half-bits, and were trained on SCOPe40 with TM-align alignments.

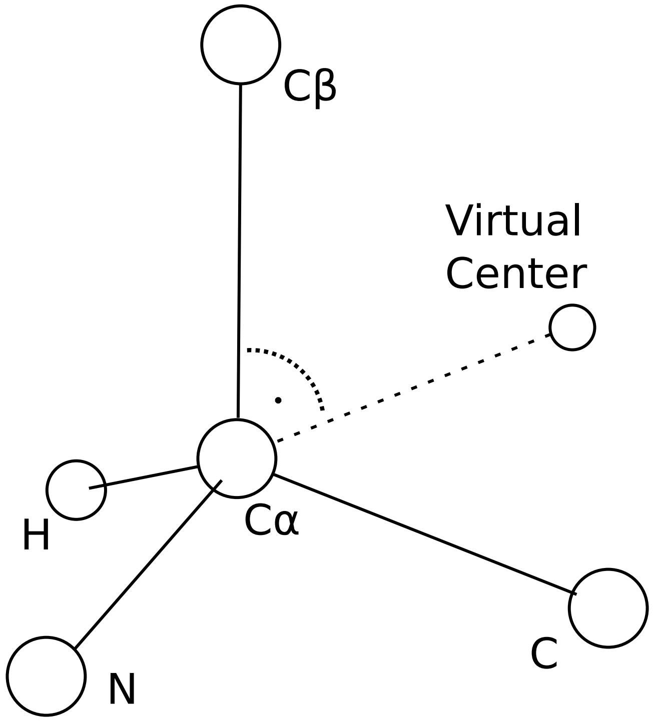

Supplementary Figure 1: **3Di virtual center.** During the transformation of structures into 3Di sequences, the virtual centers of residues are used to determine interacting residues. The optimized virtual center lies on the plane defined by the atoms N, C_α, and C_β. Moreover, C_β, C_α, and the virtual center form an angle of 90°. The distance between the virtual center and C_α equals twice the distance between C_β and C_α. For glycines, the C_β is approximated by assuming that the C_β, $C_{backbone}$, and N atoms are arranged at the vertices of a regular tetrahedron with C_α at its centroid, and a centroid to vertex distance of 1.5336 Å.

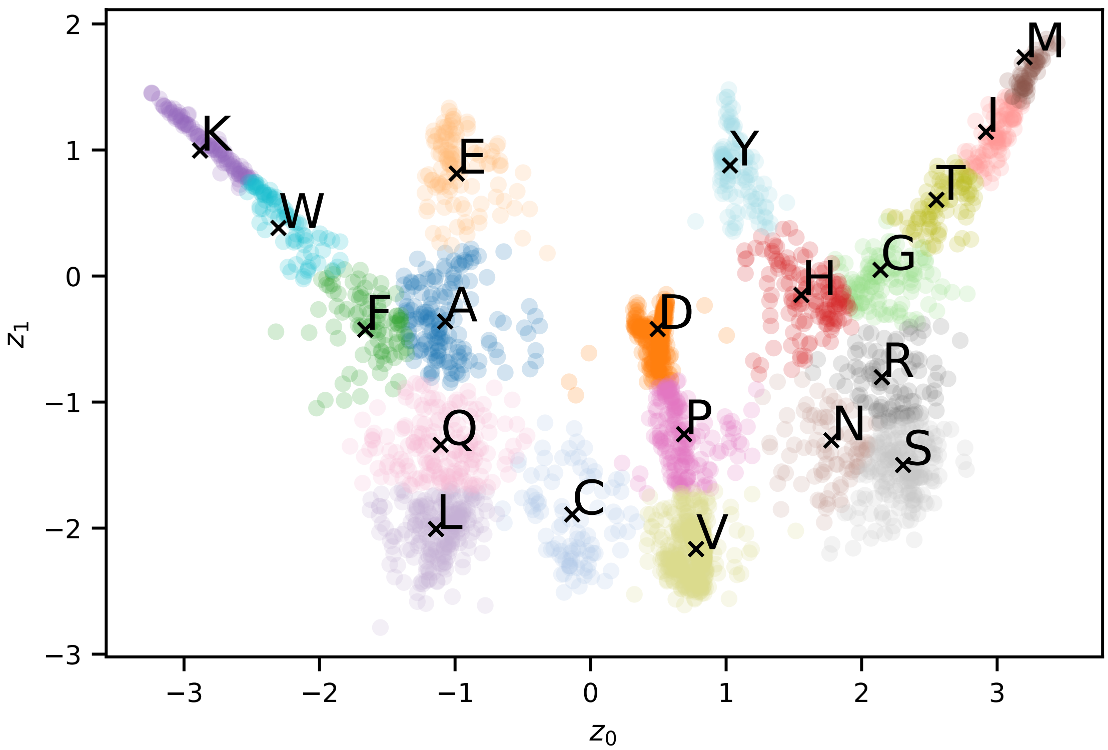

Supplementary Figure 2: **Latent space representation learned by encoder network** The encoder network of the VQ-VAE encodes the 3Di descriptor of a residue into a two-dimensional representation. Here, we show this latent space representation of 3000 sampled residues. Each circle represents a residue and is colored according to its nearest centroid (x), which discretizes the residue to a 3Di state.

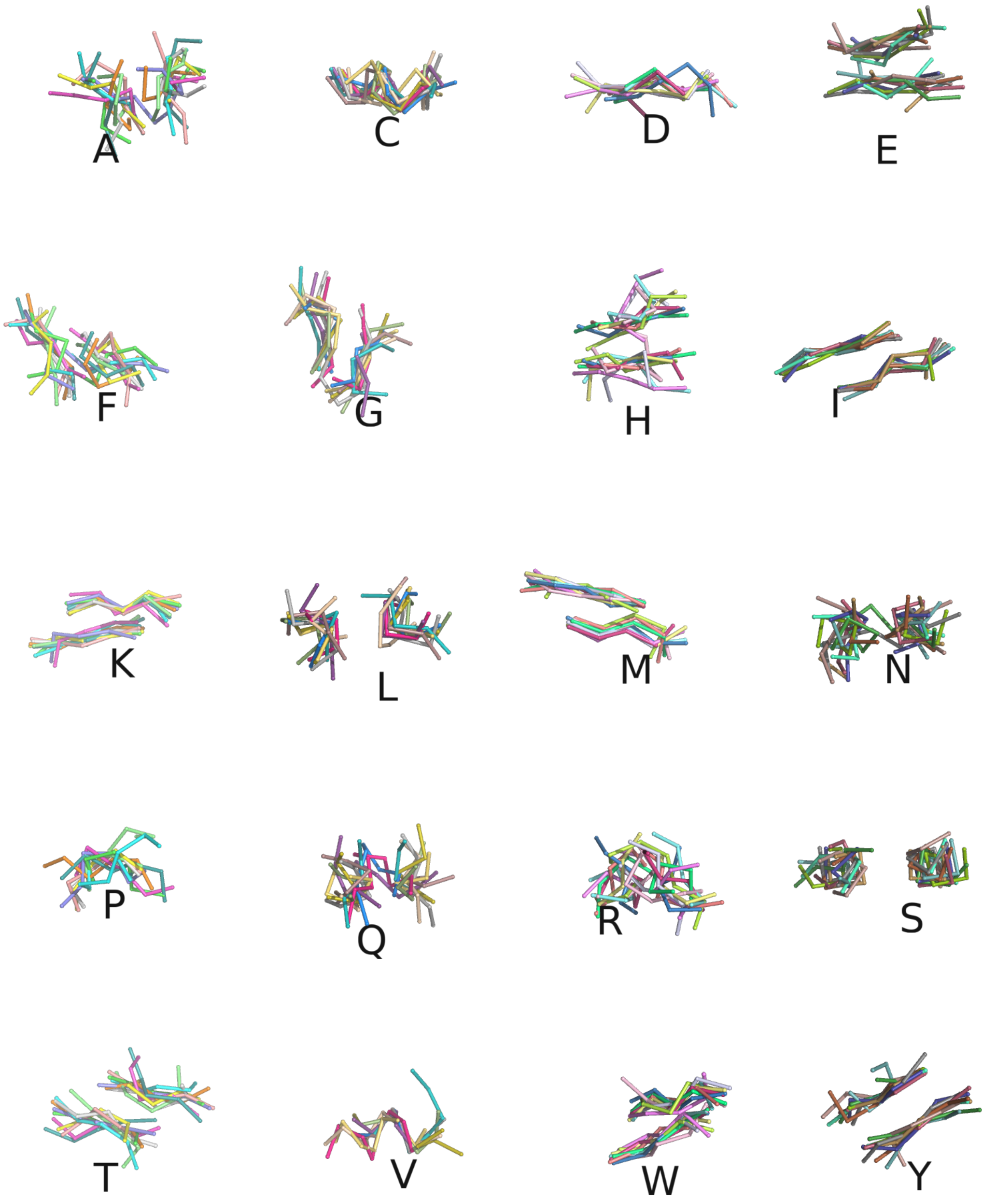

Supplementary Figure 3: **3Di state visualizations** Each 3Di state represents a conformation between two three-residue backbone fragments. To visualize this conformation, we sampled and aligned ten fragment pairs for each state, where the paired fragments have the same color. Here, five-residue fragments are shown, however the 3Di states describes only the conformation of the inner three-residue fragments.

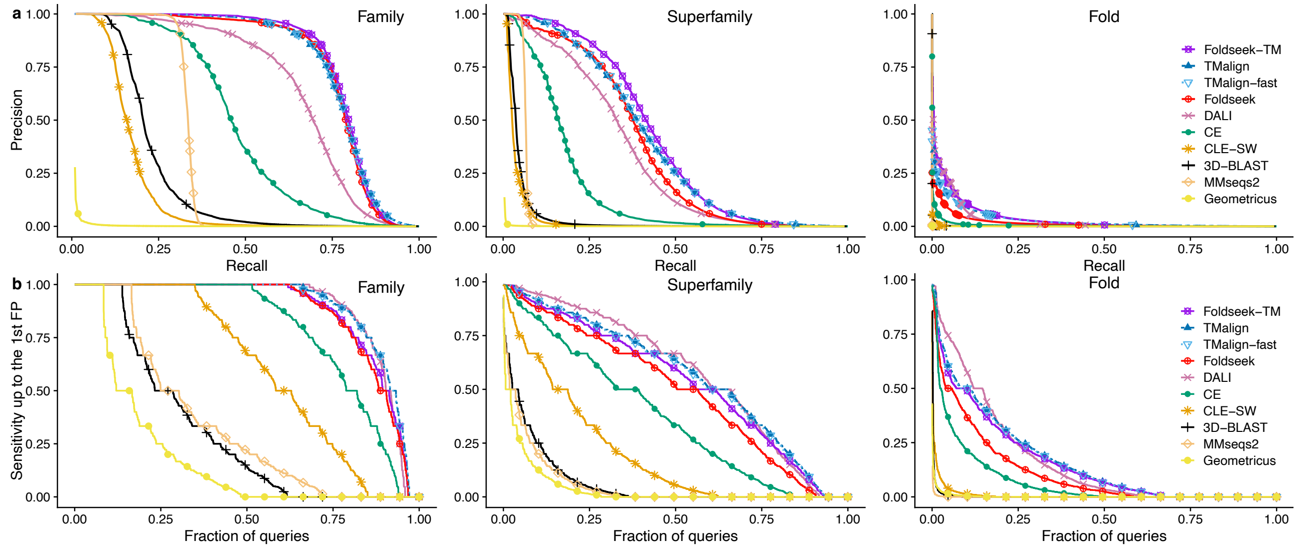

Supplementary Figure 4: **Precision-Recall curves and cumulative distributions of sensitivity for the SCOPe benchmark** We measured the sensitivity of Foldseek and seven structure alignment tools on the SCOPe40 dataset by plotting precision-recall curves and the cumulative distributions of sensitivity up to the first FP on family, superfamily, and fold level.

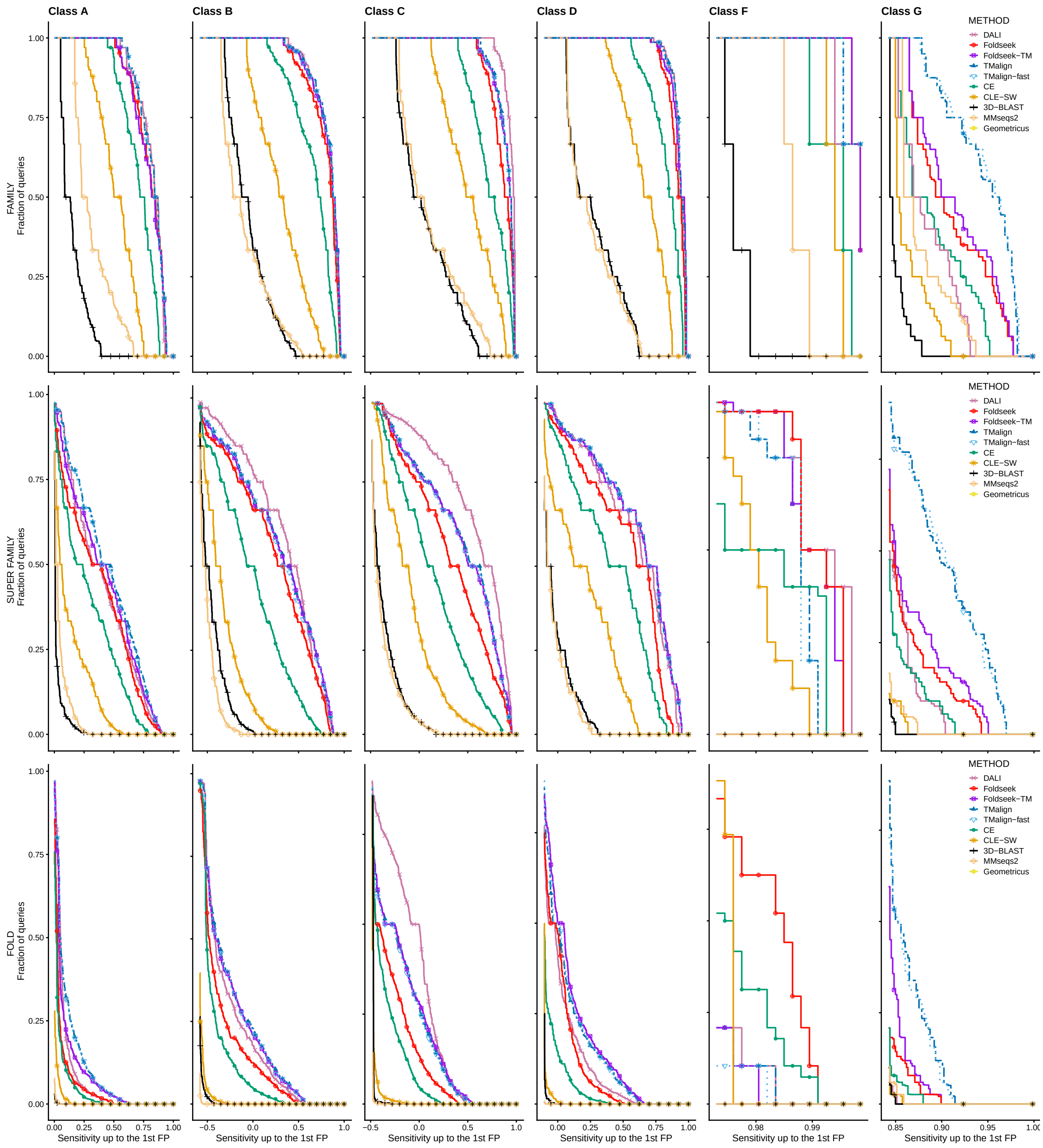

Supplementary Figure 5: **SCOPe benchmark by class.** We measured the sensitivity of Foldseek and six structure alignment tools on the SCOPe40 dataset, which are divided into six SCOPe classes: (1) Class A contains all alpha proteins, (2) class B has all beta proteins, (3) class C consists alpha-beta proteins with mainly parallel beta strands (a/b), (4) class D alpha-beta proteins with mainly antiparallel beta strands (a+b), (5) class F consists of membrane and cell surface proteins and peptides, and (6) class G contains small proteins.

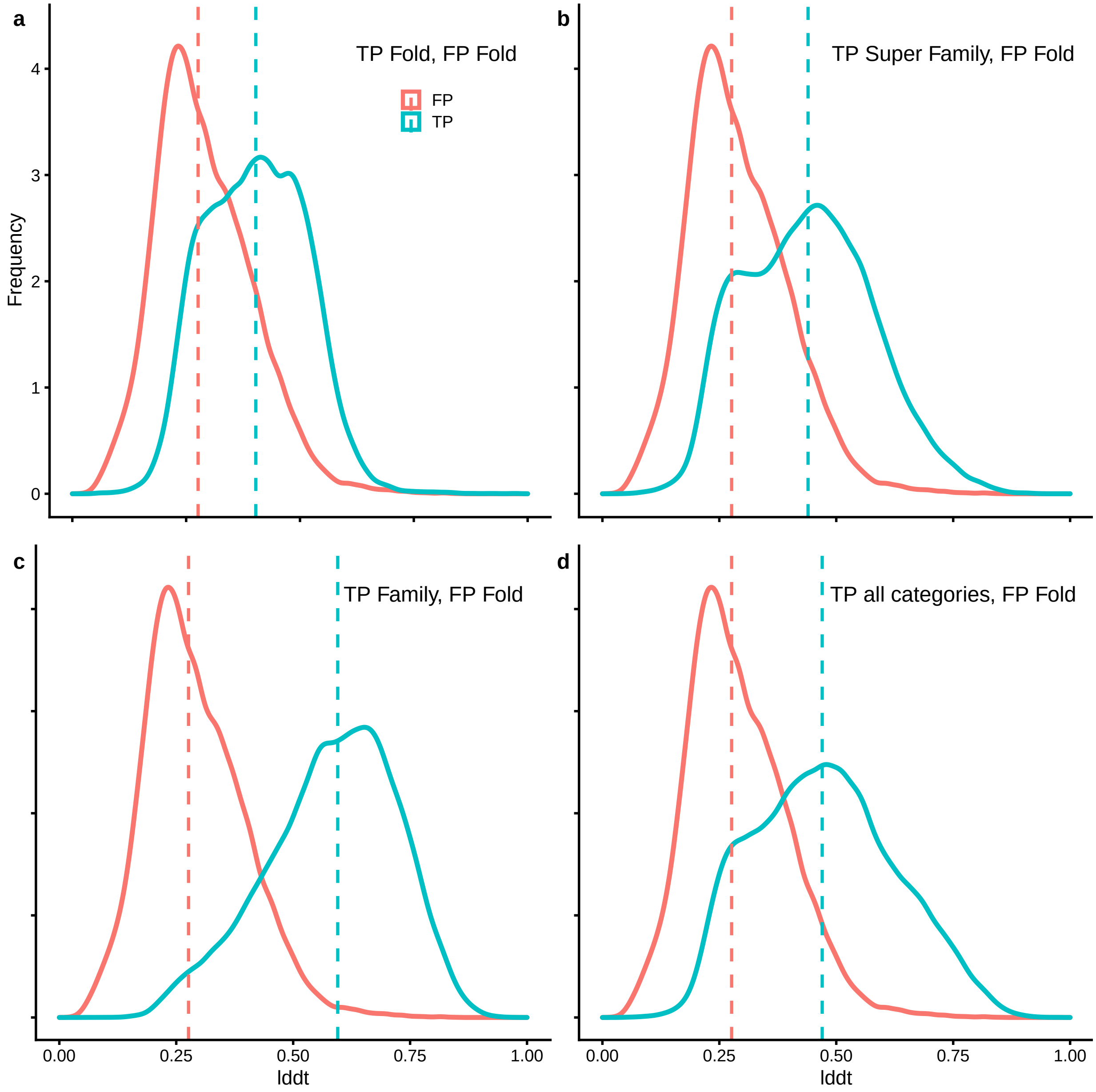

Supplementary Figure 6: SCOPe40 TP and FP average LDDT per alignment distribution. Average LDDT distribution of 10,000 randomly chosen pairwise alignments of TP and FP hits for TM-align, Foldseek, Dali, and CLE. FP shown for folds (a-d), TP shown for folds (a), superfamilies (b), families (c), and all categories together (d).

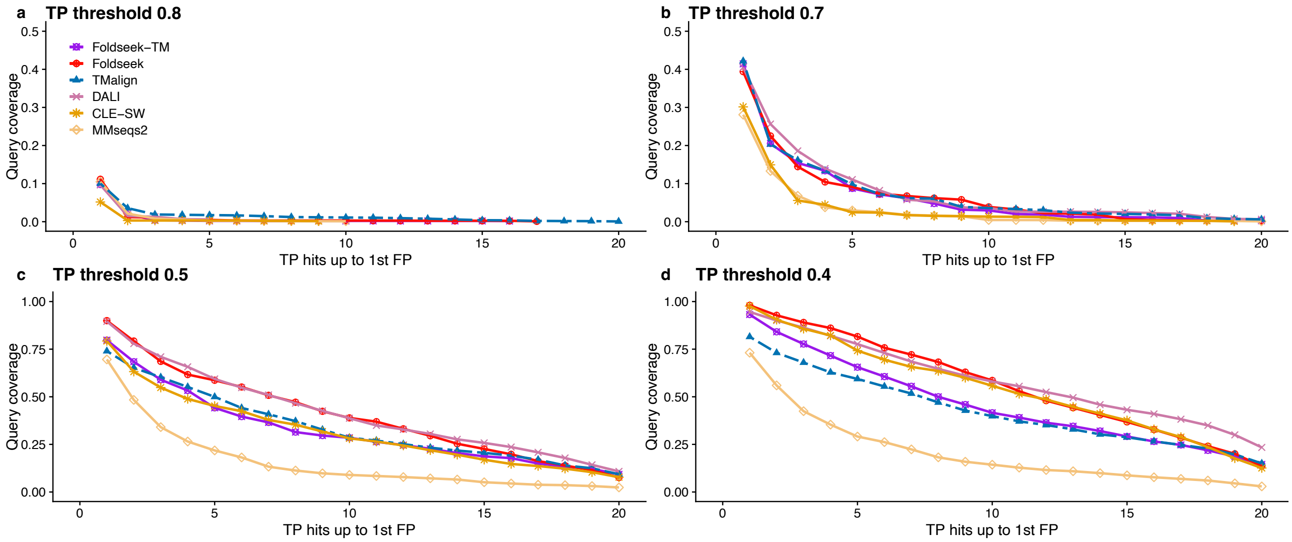

Supplementary Figure 7: **AlphaFold coverage benchmark at different TP thresholds.** This figure shows the same benchmark as in **Fig 2d**, but with different LDDT thresholds for TP hits, while the FP threshold remains at 0.25.

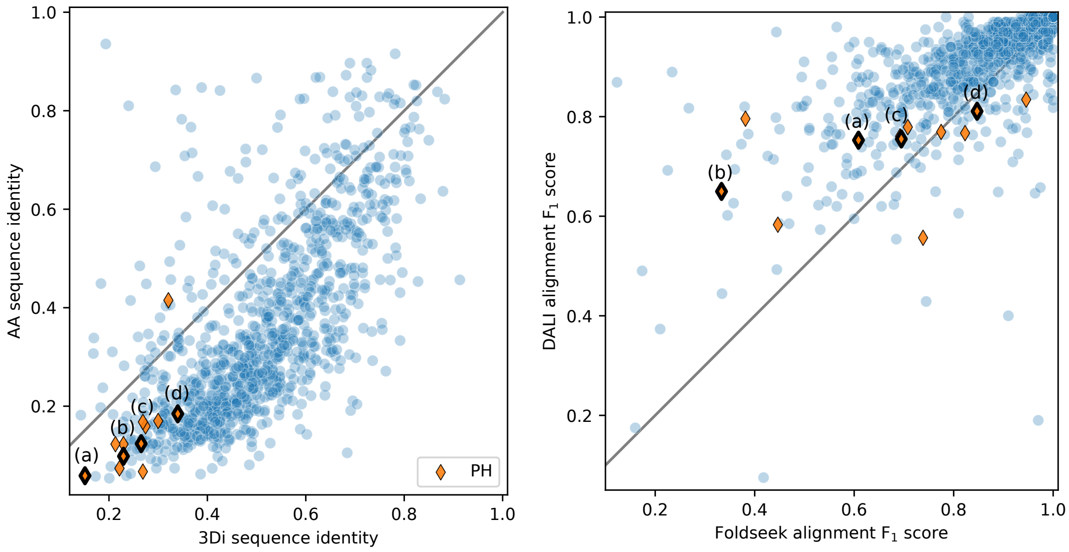

(1) **Left**: Comparison of sequence identities of amino acid (AA) and 3Di sequences in HOMSTRAD reference alignments. For each HOMSTRAD family, the alignment between the first and last member was used (blue circles). We selected the HOMSTRAD family `PH` as its members cover a large range in both amino acid sequence identity and Foldseek's alignment quality. The orange diamonds represent alignments within this family (between `1dro` and all other family members). Next, we selected a series of alignments with increasing amino acid sequence identity ((a) to (d)). For each alignment, the HOMSTRAD reference alignment and Foldseek's alignment are shown below. **Right**: Alignment quality comparison on HOMSTRAD families (blue points, also shown in **Fig. 2f**) between Foldseek and Dali, which performed best in the HOMSTRAD benchmark. For each family, the alignment quality between the first and last family member is described by the F_1 score, which is the harmonic mean of sensitivity and precision. The average sensitivity and precision for all methods is shown in **Fig. 2e**. 46 of 1032 families are not shown, because Dali did not return an alignment.

(a) 1dro - 1mai (sensitivity = 0.57, precision = 0.66, F_1 score = 0.61)

HOMSTRAD alignment: (RMSD=5.4Å for 99 residues, seq.id. = 6% (AA), 15% (3Di))

```
1dro    1 D------PDDDDDAAAWDQFWKQKFDDDDDAVPHPDHIDGATWDDAAA---KTFGARDCAVVVVDVQDHPVVTDMGGALQ
          |               |+++|++ +          +++++ +|+       |+|   |         |++ |  +++  +
1mai    1 DLVPDPLNVVQQV---WAFWWWFDAQ---------PRTHTKIWHQHP-VNFKIFIHDDDP-------DDDRVVRIDTLVF

1dro   72 W-AWDADC------DDDQSNL---KIWTGGPP-RIIIITRDDDNVVRVVSVVGRHCRNN
          + |+++++      +         ++||+|++   |++++++|+| ++   | + ++|
1mai   61 FDAKAFAQPDPRCVHRVPVADRLFWMWTDGDDPDDITTIGHPDSVNSCSVRVVSVVSND
```

Foldseek alignment: (RMSD=5.5Å for 85 residues)

```
1dro   11 WDQFWKQKFDDDDDAVPHPDHIDGATWDDAA--AKTFGARDCAVVVVDVQDHPVVTDMGGALQW-AWDADCDD-------
          |+++|++      || |   ++++ +|+     +|+|   |      |  |+ ||  +++  ++ |++++++|
1mai   14 WAFWWWF------DAQP---RTHTKIWHQHPVNFKIFIHDD------DPDDDRVVR-IDTLVFFDAKAFAQPDPRCVHRV

1dro   81 --DQSNLKIWTGG--PPRIIIITRDDDNVVRVVSVV
            + +  ++||+|  |+ |++|+ ++|+| ++   |
1mai   78 PVADRLFWMWTDGDDPDDITTIG-HPDSVNSCSVRV
```

(2)

(b) 1dro - 1b55A (sensitivity = 0.32, precision = 0.35, F_1 score = 0.33)

HOMSTRAD alignment: (RMSD=5.6Å for 100 residues, seq.id. = 10% (AA), 23% (3Di))

```
1dro        6 DDAAAWDQFWKQKFDDDDDAVPHPDHIDGATWDDAAA-KTFGARD-CAVVVVDVQDHPVVTDMGGALQW-AWDADC----
              ||  +++ +|+    +|+    ++ +||+ +| |+   ++++|++     | |  + +   |+++ +|| ++| +
1b55a       1 DDWPDKAWWWWWDPCPDP---VDDTDIDTWIWTDDL-FWIWTAHADPV--VGDGHHTD---DIDTLQQWQDKDWAPFDDD

1dro       79 DDDQSNL--KIWTGGPP--------------------RIIIITRDDDNVVRVVSVVGRHCRNNDD---------------
              || |                                   |||+ + | || ||  | + ++  ++
1b55a      72 DDQQQDDDD--------DPDVVQQRRRFNTWMWTAGDVAIIIITGSDPVVVVVVRVVSCVSHVPHDRHDQAAARYDQPDQ

1dro      122 -------------------D
                                 |
1b55a     144 GGPVVRDRRRHDGHPDGDDD
```

Foldseek alignment: (RMSD=6.7Å for 92 residues)

```
1dro       14 FWKQKFDDDDDAVPHPDHIDGATWDDAAAKTFGARDCAVVVVDVQDHPVVT-DMGGALQW-AWD---ADCDDDQS-----
              +|+ +  |+ ++  ++ +||+ +| |+ +++++|+  |  ||+  +|   | |+++ +|| ++|   +| ||||
1b55a       8 WWWWW--DPCPDPVDDTDIDTWIWTDDLFWIWTAH--ADPVVGDGHH---TDDIDTLQQWQDKDWAPFDDDDDQQQDDDD

1dro       84 -----------NLKIWTGGPPRIIIITRDDDNVV---RVVSVV
                         | ++|| |+  |||||+ |+ ||   ||||+|
1b55a      81 DPDVVQQRRRFNTWMWTAGDVAIIIITGSDPVVVVVVRVVSCV
```

(3)

(c) 1dro - 1dyna (sensitivity = 0.65, precision = 0.74, F_1 score = 0.69)

HOMSTRAD alignment: (RMSD=4.3Å for 101 residues, seq.id. = 12% (AA), 27% (3Di))

```
1dro        6 DDAAAWDQFWKQKFDDDDDAVPHPDHIDGATWDDAAA-KTFGARDCAVVVVDVQDHPVVTDMGGALQWAWDADC-DDDQS
              ||  +++ +|+|  |        | |+|  +| |+   ++++|+|+        + |   |+ +  |+|+ ++  ||+
1dyna       1 DDWLDKAKWWFQQDD------VVPVHTDIWIWIDDL-FWIWTANDVVRP-----GIP---DIFTLAQKAKEFPPCDDPDQ

1dro       84 NLKIWTGGPP-------RIIIITRDDDNVVRVVSVVGRHCRNNDD
                |+|+ + |       + | |+ | | || ||  | + ++
1dyna      66 WGKMWIARNPDQARPDNHSIGIITDNDPVVVVVVQVSSVVSPHYY
```

Foldseek alignment: (RMSD=4.2Å for 89 residues)

```
1dro       10 AWDQFWKQKFDDDDDAVPHPDHIDGATWDDAAAKTFGARDCAVVVVDVQDHPVVT-DMGGALQWAW-DADCDDDQSNLKI
              +++ +|+|      |+|    |+|  +| |+ +++++|+|+      | ++   + |+ +  |+|+   +|||+    |+
1dyna       5 DKAKWWFQ-----QDDVVPV-HTDIWIWIDDLFWIWTANDV------VRPG---IPDIFTLAQKAKEFPPCDDPDQWGKM

1dro       88 WTGGPP--------RIIIITRDDDNVVRVVSVVGR
              |+ + |        +| ||| | | || ||  | +
1dyna      70 WIARNPDQARPDNHSIGIIT-DNDPVVVVVVQVSS
```

(4)

(d) 1dro - 1qqgA (sensitivity = 0.77, precision = 0.94, F_1 score = 0.85)

HOMSTRAD alignment: (RMSD=3.4Å for 94 residues, seq.id. = 18% (AA), 34% (3Di))

```
1dro       7 DAAAWDQFWKQKFDDDDDAVPHPDHIDGATWDDAAA-------KTFGARDCAVVVVDVQDHPVVTDMGGALQW-AWDADC
             | ++|| +  |  +             + ++  +||       ++| ++|  +|||   | |   |+|+  |+ +||+|+
1qqga      1 DWDDWDWWAQQ--P----------VRQTWIKTFDAADPPPHATWIFIDNDDVCVVVVPDDTP---DIGHLVQFPDWDFDQ

1dro      79 DDDQSNLKIWTGGPP-RIIIITRDDDNVVRVVSVVGRHCRN
             |+   + ||||+||    | |++++| ||+||  | + +|
1qqga     66 DPR-ARTKIWTHGP-P-DIGIIGHPDVVVSVVVSVSSVVRD
```

Foldseek alignment: (RMSD=3.2Å for 76 residues)

```
1dro      34 GATWDDAAA-------KTFGARDCAVVVVDVQDHPVVTDMGGALQW-AWDADCDDDQSNLKIWTGGPPRIIIITRDDDNV
             + ++  +||       ++| ++|  +|||   | |   |+|+  |+ +||+|+   + + ||||+||| |  |++++| |
1qqga     16 TWIKTFDAADPPPHATWIFIDNDDVCVVVVPDDTP---DIGHLVQFPDWDFDQDPRA-RTKIWTHGPPDI-GIIGHPDVV

1dro     106 VRVVSVVGR
             |+||  | +
1qqga     91 VSVVVSVSS
```

(5)

Supplementary Figure 8: **Foldseek alignment quality of example alignments** Subfigure (1) shows the sequence identities and alignment qualities of the four selected alignments in context of all HOMSTRAD alignments. The alignments are shown in subfigures (2) to (5). For each example alignment, the 3Di sequences aligned by HOMSTRAD and by Foldseek are shown. The sequence identities of aminco acid (AA) and 3Di sequence in the HOMSTRAD alignment are provided. The root mean square deviation (RMSD) was calculated for both alignments. The symbol | denotes an alignment of identical 3Di states, and the symbol + denotes an alignment between 3Di states with positive substitution score.

	Query	Target	Foldseek		TM align
			Bit per column	E-value	TM score
**Model**	AF-P0AEC3-F1	AF-P30855-F1	3.021	3.459E-43	0.291
**Protein Function**	Aerobic respiration control sensor protein ArcB	Sensor protein EvgS			
	EC: 2.7.13.3	EC: 2.7.13.3			
**Structure**	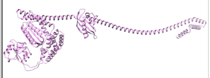	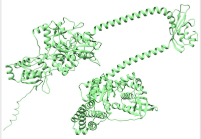			
**Sequence Length**	778	1197			
**Avg. pLDDT**	81.193	82.329			
**Predicted Aligned Error**	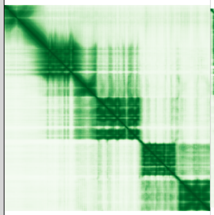	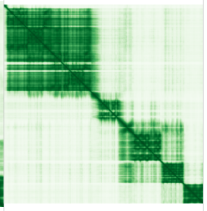			
**Model**	AF-Q4CPQ6-F1	AF-Q4CLF8-F1	5.856	4.293E-61	0.233
**Protein Function**	Calpain cysteine peptidase, putative	Calpain-like cysteine peptidase, putative			
	-	-			
**Structure**	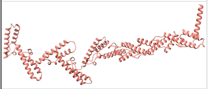	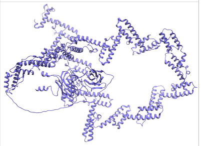			
**Sequence Length**	1753	631			
**Avg. pLDDT**	82.150	89.192			
**Predicted Aligned Error**	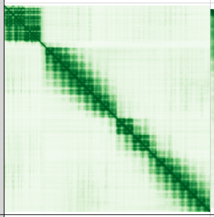	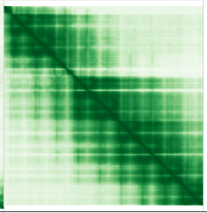			
**Model**	AF-Q54P41-F1	AF-Q54QK8-F1	1.938	1.146E-38	0.367
**Protein Function**	EGF-like domain-containing protein	EGF-like domain-containing protein			
	-	-			
**Structure**	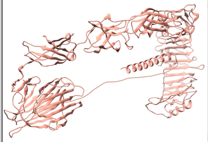	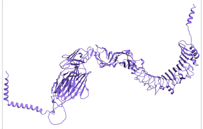			
**Sequence Length**	992	1064			
**Avg. pLDDT**	80.493	86.856			
**Predicted Aligned Error**	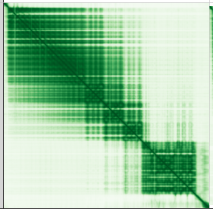	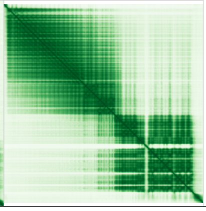			

Supplementary Table 3: **Outliers in Foldseek's top hits in AlphaFoldDB** Three hand-picked outliers from the scatter plot in **Supplemental Fig. 9** are shown here. The outliers were selected from examples where Foldseek has a hit with a score greater 1.0 bits per column and an TM-align score below 0.5. These three models taken from the AlphaFoldDB contain well predicted local structures (with high average pLDDTs of over 80). However, the global structure is not well predicted (visible in the light green areas of the Predicted Aligned Error). This shows Foldseek's ability to detect remote homologies that are difficult to find by TM-align's global alignment.

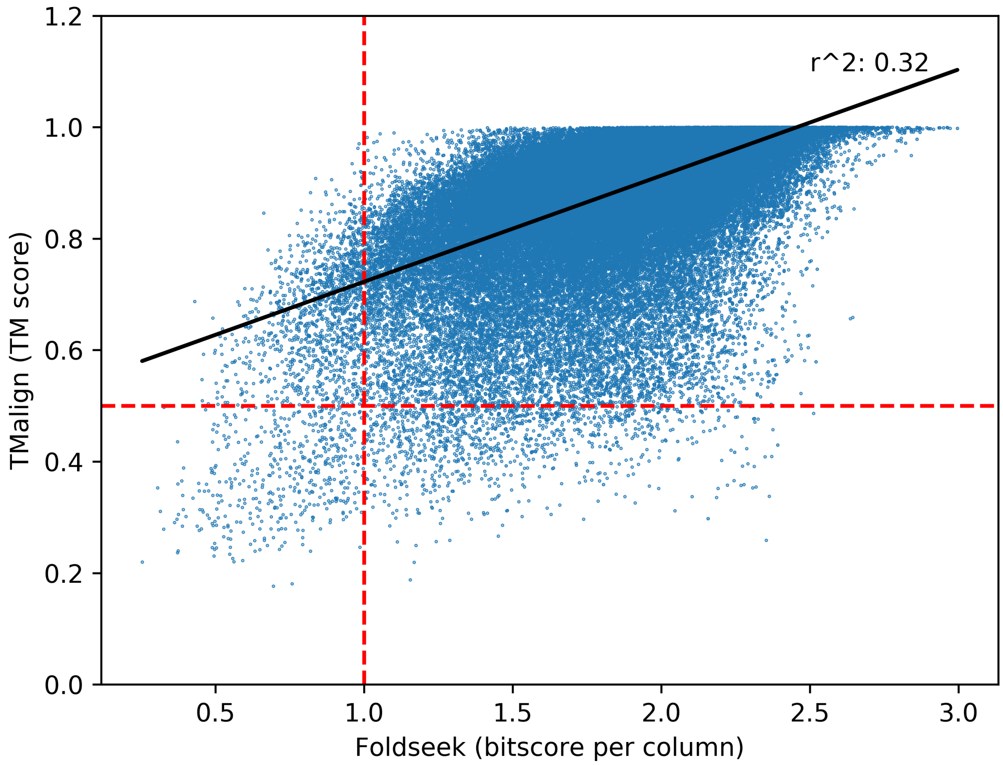

Supplementary Figure 9: Structural similarity of Foldseek's top hits in AlphaFoldDB After running an all-versus-all alignment on AlphaFoldDB with Foldseek, the top hit (sorted by Foldseek's E-value) for each query was collected and structural similarity was calculated by TM-score as a comparison. The thresholds for each tool (Foldseek: > 1.0 bits per column, and TM-align: > 0.5 TM-score) are shown by red-dashed lines.

Foldseek

Model	Description	Homologous	Probability
AF-P03876-F1-v1	PUTATIVE COX1/OXI3 INTRON 2 PROTEIN	✓	1.0
AF-P03875-F1-v1	PUTATIVE COX1/OXI3 INTRON 1 PROTEIN	✓	1.0
AF-P0A3U1-F1-v1	GROUP II INTRON-ENCODED PROTEIN LtrA	✓	1.0
AF-A0A0G2L2B6-F1-v1	REVERSE TRANSCRIPTASE DOMAIN-CONTAINING PROTEIN	✓	1.0
AF-Q54ZK5-F1-v1	REVERSE TRANSCRIPTASE DOMAIN-CONTAINING PROTEIN	✓	1.0
AF-Q55D49-F1-v1	REVERSE TRANSCRIPTASE DOMAIN-CONTAINING PROTEIN	✓	1.0
AF-Q54E03-F1-v1	REVERSE TRANSCRIPTASE DOMAIN-CONTAINING PROTEIN	✓	1.0
AF-P11369-F1-v1	LINE-1 RETROTRANSPOSABLE ELEMENT ORF2	✓	1.0
AF-P0A3U0-F1-v1	GROUP II INTRON-ENCODED PROTEIN LtrA	✓	1.0
AF-B1N1A3-F1-v1	PUTATIVE NICOTINE OXIDOREDUCTASE	✓	1.0

Foldseek-TM

Model	Description	Homologous	TM score
AF-A0A1D6L213-F1-v1	RETROVIRUS-RELATED POL POLYPROTEIN LINE-1	✓	0.501
AF-A0A1D8PFY7-F1-v1	REVERSE TRANSCRIPTASE DOMAIN-CONTAINING PROTEIN	✓	0.484
AF-A0A0R0J4L8-F1-v1	RNA-DEPENDENT RNA POLYMERASE	✓	0.477
AF-A0A1D6Q028-F1-v1	RETROVIRUS-RELATED POL POLYPROTEIN LINE-1	✓	0.474
AF-A0A1D6ERU1-F1-v1	REVERSE TRANSCRIPTASE DOMAIN-CONTAINING PROTEIN	✓	0.465
AF-Q54LM7-F1-v1	REVERSE TRANSCRIPTASE DOMAIN-CONTAINING PROTEIN	✓	0.465
AF-E7F608-F1-v1	REVERSE TRANSCRIPTASE	✓	0.465
AF-Q2QZT5-F1-v1	REVERSE TRANSCRIPTASE	✓	0.459
AF-P38478-F1-v1	UNCHARACTERIZED MITOCHONDRIAL PROTEIN YMF40	✓	0.457
AF-Q47688-F1-v1	PROTEIN YkfC	✓	0.456

TMalign

Model	Description	Homologous	TM score
AF-A0A1D6L213-F1-v1	RETROVIRUS-RELATED POL POLYPROTEIN LINE-1	✓	0.501
AF-A0A1D8PFY7-F1-v1	REVERSE TRANSCRIPTASE DOMAIN-CONTAINING PROTEIN	✓	0.484
AF-A0A0R0J4L8-F1-v1	RNA-DEPENDENT RNA POLYMERASE	✓	0.477
AF-A0A1D6JF34-F1-v1	RETROVIRUS-RELATED POL POLYPROTEIN LINE-1	✓	0.474
AF-A0A1D6Q028-F1-v1	RETROVIRUS-RELATED POL POLYPROTEIN LINE-1	✓	0.473
AF-A0A1D6G0V4-F1-v1	RETROVIRUS-RELATED POL POLYPROTEIN LINE-1	✓	0.473
AF-A0A1D6H1V1-F1-v1	RETROVIRUS-RELATED POL POLYPROTEIN LINE-1	✓	0.473
AF-A0A1D6LAQ1-F1-v1	RETROVIRUS-RELATED POL POLYPROTEIN LINE-1	✓	0.473
AF-A0A1D6K060-F1-v1	RETROVIRUS-RELATED POL POLYPROTEIN LINE-1	✓	0.472
AF-A0A1D6ERU1-F1-v1	REVERSE TRANSCRIPTASE DOMAIN-CONTAINING PROTEIN	✓	0.466

Dali

Model	Description	Homologous	Z-score
AF-P92543-F1	UNCHARACTERIZED MITOCHONDRIAL PROTEIN ATMG01110	✓	11.7
AF-Q3EC48-F1	MITOVIRUS RNA-DEPENDENT RNA POLYMERASE	✓	11.0
AF-K7LVI8-F1	UNCHARACTERIZED PROTEIN	✓	10.3
AF-K7LE20-F1	UNCHARACTERIZED PROTEIN	✓	10.2
AF-P03876-F1	PUTATIVE COX1/OXI3 INTRON 2 PROTEIN	✓	10.2
AF-Q62120-F1	TYROSINE-PROTEIN KINASE JAK2	✓	9.9
AF-K7K434-F1	UNCHARACTERIZED PROTEIN	✓	9.8
AF-A0A1D6P530-F1	PROTEIN KINASE DOMAIN CONTAINING PROTEIN	✓	9.6
AF-A0A1D6GQJ0-F1	NON-SPECIFIC SERINE/THREONINE PROTEIN KINASE	✓	9.6
AF-Q54E07-F1	REVERSE TRANSCRIPTASE DOMAIN-CONTAINING PROTEIN	✓	9.6

Supplementary Table 4: **RdRp search** The search for homologous structures of RdRps (6M71_A) was done with Foldseek, Foldseek-TM module and TM-align against the AlphaFold/Proteome and AlphaFold/Swiss-Port database. Dali was only searched through the AlphaFold/Proteome database due its long runtime. The top 10 hits of the respective method are shown. We labeled if structures are homologous by manually checking the Uniprot website for RdRp/RT or Kinase domains.

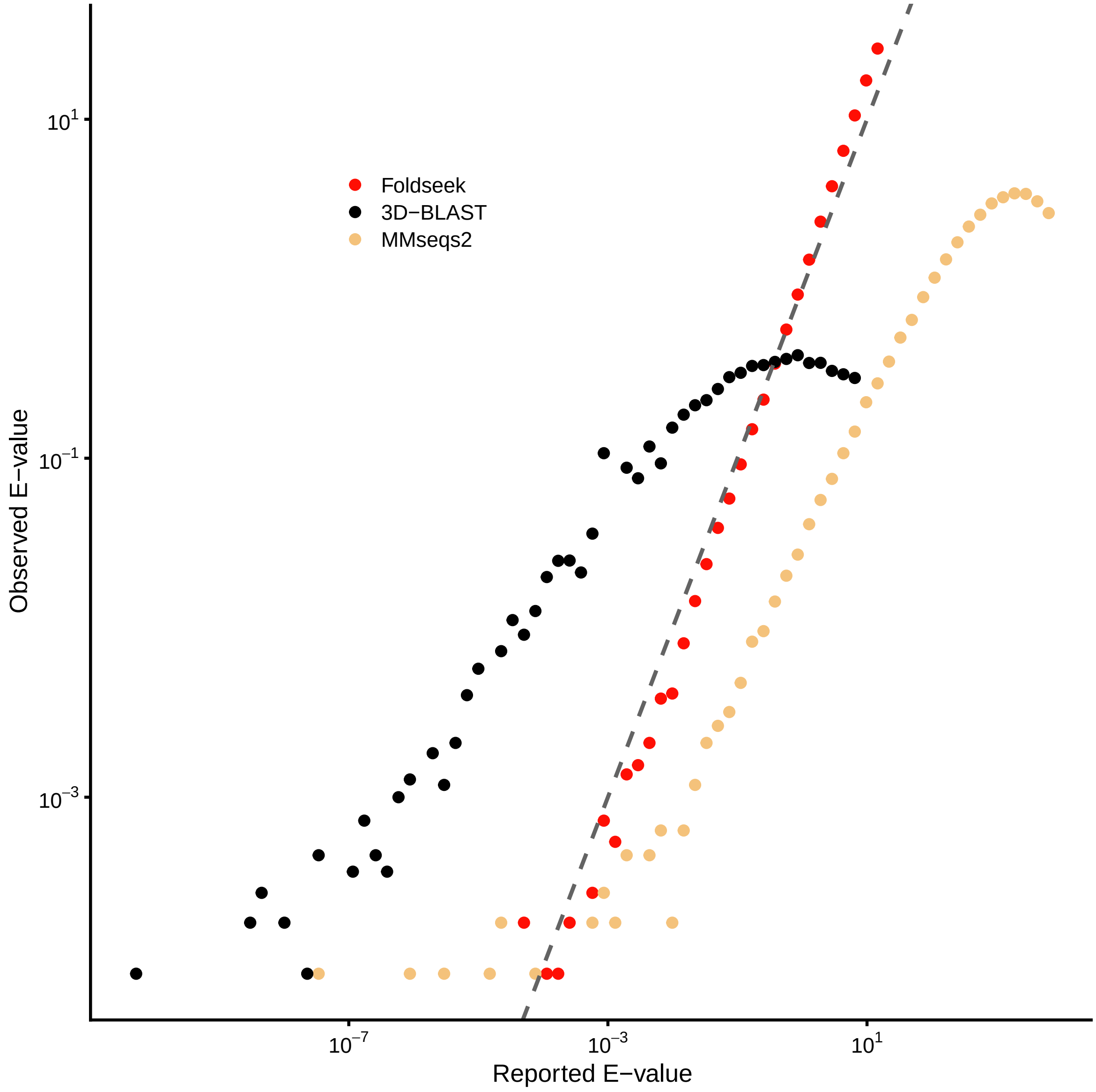

Supplementary Figure 10: **Accuracy of reported E-values** Mean number of FPs per query below the reported E-value threshold measured on SCOP (see **Supplementary Fig. 12** for AlphaFold DB structures)

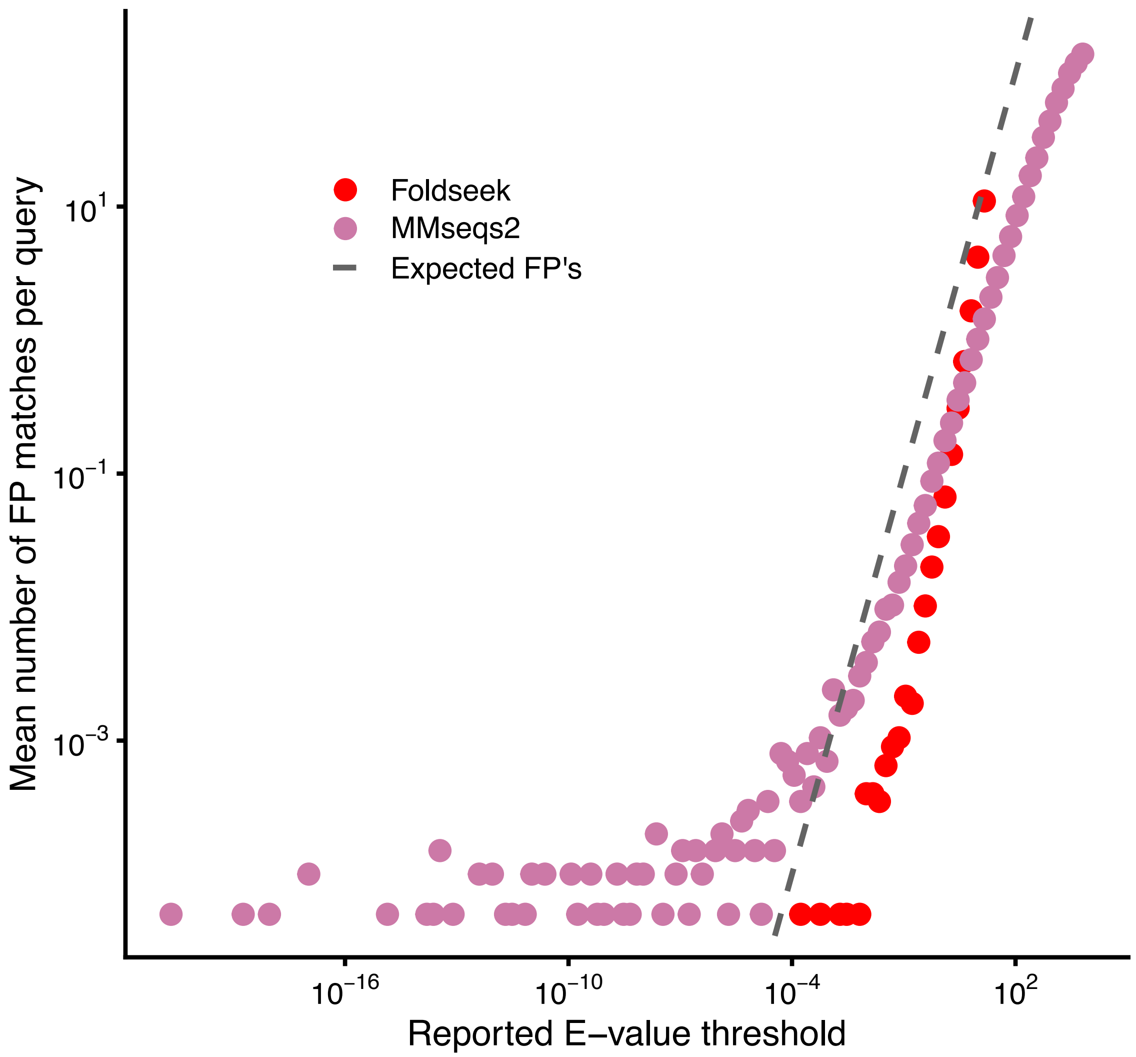

Supplementary Figure 11: **E-values for shuffled sequences from AlphaFoldDB** As described in the Methods section, we trained a neural network to predict the Gumbel distribution parameters from the input sequence composition. Here, we show the mean number of FP matches per query versus the reported E-value threshold for 100 000 randomly sampled sequences from the AlphaFoldDB and shuffled those as described earlier. E-values are shown for Foldseek and MMseqs2.

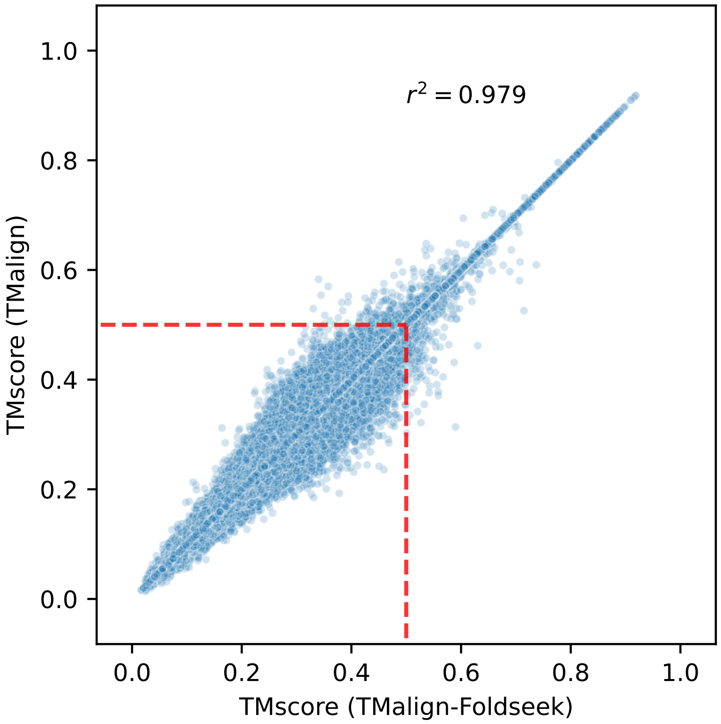

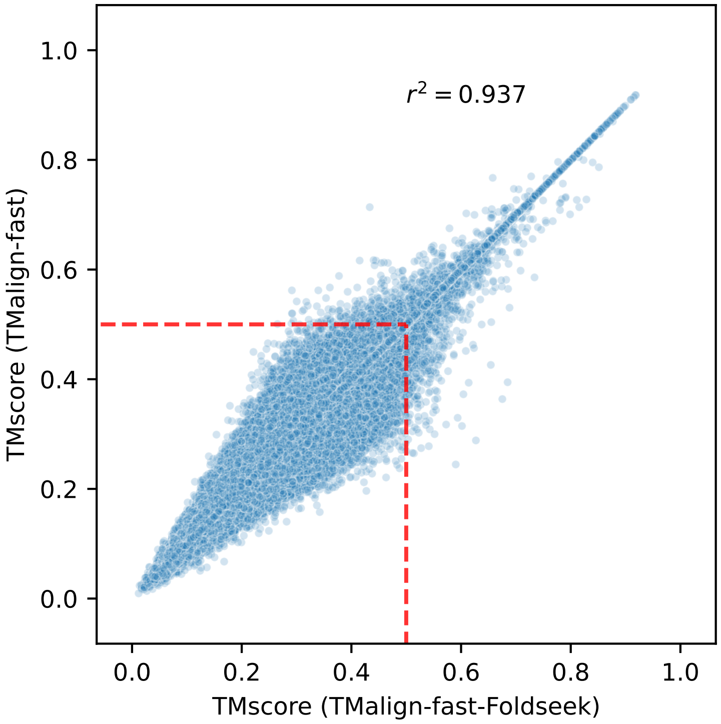

Supplementary Figure 12: **TMalign vs TMalign-Foldseek Benchmark** To compare our TMalign version (TMalign-Foldseek) with the original TMalign version, we sampled 1000 SCOPe domains and performed an all-vs-all alignment in both TMalign modes (normal and fast). TMalign-Foldseek was 1.7 times faster (253 minutes) compared to TMalign (438 minutes). When using the fast mode, this factor remained the same between TMalign-Foldseek (75 minutes) and TMalign (131 minutes). The two figures show the relation between the TMscores of the two implementations in both normal and fast mode respectively. Note that TMscores below 0.5 (red dashed line) indicate that the structures are not from the same fold.

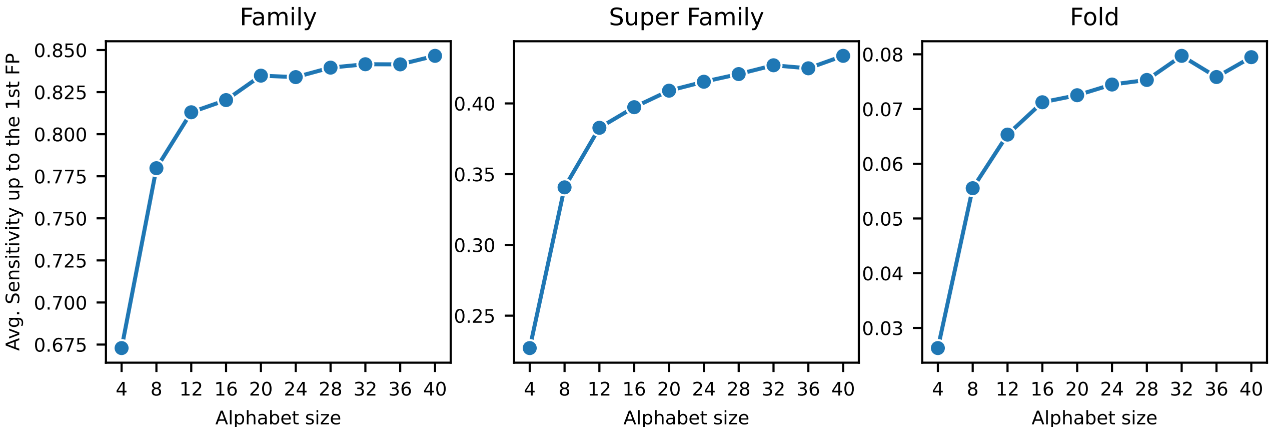

Supplementary Figure 13: **SCOPe benchmark sensitivity by 3Di alphabet size** We evaluated the effect of the alphabet size by training a new alphabet (VQ-VAE and substitution matrix) and testing it on the SCOPe benchmark for each size. The average sensitivity up to the 1st FP is the area under the ROC curve, as in the benchmark in **FIG. 2**.

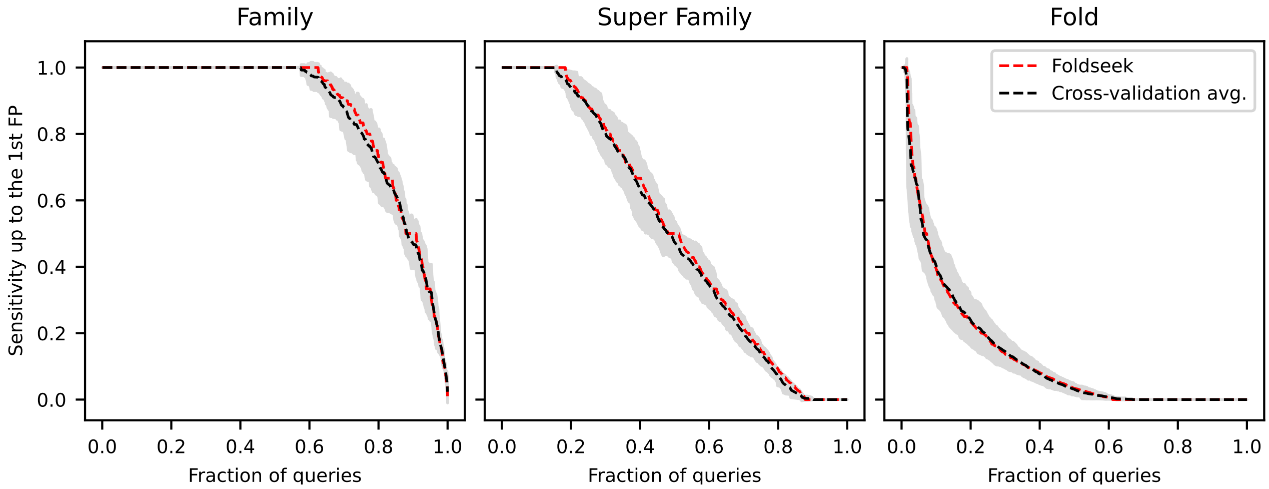

Supplementary Figure 14: **3Di alphabet: 4-fold cross-validation on SCOPe40** The 3Di alphabet was trained and benchmarked on the same dataset (SCOPe40). We therefore tested the generalization capabilities of the model with a 4-fold cross-validation, where the SCOPe40 dataset was split by fold into four parts (all domains of each fold are in the same split). Four new models were trained on three parts and benchmarked on the remaining part. The figure shows their mean sensitivity (black) and the standard deviation (gray area) in comparison to the final 3Di alphabet that is used in Foldseek (red), which was trained on the entire dataset.
